# Evaluation of the immunomodulatory effects of cobalt, copper and magnesium ions in a pro inflammatory environment

**DOI:** 10.1038/s41598-021-91070-0

**Published:** 2021-06-03

**Authors:** Leire Díez-Tercero, Luis M. Delgado, Elia Bosch-Rué, Roman A. Perez

**Affiliations:** Bioengineering Institute of Technology, Universitat Internacional de Catalunya, Sant Cugat del Vallès, Barcelona Spain

**Keywords:** Inflammation, Biomedical engineering

## Abstract

Biomaterials and scaffolds for Tissue Engineering are widely used for an effective healing and regeneration. However, the implantation of these scaffolds causes an innate immune response in which the macrophage polarization from M1 (pro-inflammatory) to M2 (anti-inflammatory) phenotype is crucial to avoid chronic inflammation. Recent studies have showed that the use of bioactive ions such as cobalt (Co^2+^), copper (Cu^2+^) and magnesium (Mg^2+^) could improve tissue regeneration, although there is limited evidence on their effect on the macrophage response. Therefore, we investigated the immunomodulatory potential of Co^2+^, Cu^2+^ and Mg^2+^ in macrophage polarization. Our results indicate that Mg^2+^ and concentrations of Cu^2+^ lower than 10 μM promoted the expression of M2 related genes. However, higher concentrations of Cu^2+^ and Co^2+^ (100 μM) stimulated pro-inflammatory marker expression, indicating a concentration dependent effect of these ions. Furthermore, Mg^2+^ were able to decrease M1 marker expression in presence of a mild pro-inflammatory stimulus, showing that Mg^2+^ can be used to modulate the inflammatory response, even though their application can be limited in a strong pro-inflammatory environment.

## Introduction

The development of tissue engineered scaffolds has been advocated to enhance the wound healing and tissue regeneration when the native regenerative capacity of the tissue is exceeded. Currently, one of the strategies to develop new scaffolds focuses on mimicking the natural wound healing response, which starts with an acute inflammatory response, followed by the formation of new tissue due to the production of new extracellular matrix (ECM) and the migration of different cell types such as endothelial cells, fibroblasts or tissue specific stem cells^1,2^. Recently, among the different phases of wound healing, several studies have shown that the correct development and understanding of the inflammatory phase, and more precisely of macrophage response, is crucial to achieve a successful tissue regeneration^2,3^. Interestingly, different biomaterial properties such as chemical signals, topography or stiffness have shown to modify the macrophage response in cartilage^4^, bone^5^ and hernia^6^. Depending on the environmental cues, recruited macrophages can differentiate in different phenotypes, which have been classified in several subsets. Classically activated macrophages, known as the M1 phenotype, appear in response to pro-inflammatory stimuli and release pro-inflammatory mediators and cytokines, while M2 or alternatively activated macrophages are able to release anti-inflammatory cytokines that regulate the inflammatory response and modulate tissue regeneration^7^. Therefore, stimulating the resolution of the inflammatory response inducing the M2 macrophage phenotype could improve the integration of the scaffolds.

In order to modulate the inflammatory phase, biomaterials can be designed with several intrinsic and extrinsic cues to favor the M2 phenotype^8^. The tuning of biomaterials with intrinsic cues has mainly focused on the patterning of surfaces with structures that favor cell elongation, which has been shown to favor the expression of M2 phenotype^9,10^. Nevertheless, these intrinsic factors are generally applied as surface treatments in 2D systems. The complexity of including these immunomodulatory nanopatterns into 3D scaffolds has favored another approach that can modulate the immune response, which is the release of extrinsic factors. In this sense, anti-inflammatory cytokines such as IL-4 or IL-10 with adenoviral vectors and DNA plasmids or the use of siRNA targeting molecular pathways that inhibit the pro-inflammatory response have been proposed with interesting in vitro results^11–13^. However, these methods are far from clinical application due to risk for insertional mutagenesis and cytotoxicity as well as long term safety. As an alternative, bioactive ions such as cobalt, copper and magnesium are stable cues which are present at low concentrations in the body and have already shown benefits on tissue regeneration when delivered from scaffolds; however, there are limited evidences showing their immunomodulatory potential^14^.

Several studies have shown that cobalt ions were able to induce new blood vessel formation, as well as to improve wound closure and avoid bacterial infection^15^. Furthermore, copper and magnesium could stimulate osteogenesis and matrix mineralization *in vitro*^16,17^. Magnesium also improved chondrocyte differentiation when it was released from a scaffold, whereas copper, in the context of dermal wound healing, promoted skin cell migration and angiogenesis^18,19^. However, the therapeutic concentrations of these ions required to modulate macrophage response are still unclear, as well as the phenotype they are able to induce. Recent studies have shown that relatively high concentrations of Co^2+^ and Cu^2+^ ions (above 100 μM) increased the expression of M1-related markers^20,21^. Nevertheless, releasing Cu^2+^ from biomaterials could induce the M2 phenotype, suggesting that macrophage phenotype could be dependent on the concentration of the ion^22^. Recent reports have also suggested that Mg^2+^ could have an immunomodulatory potential, since an increase in M2 related markers was observed^23^. Interestingly, certain levels of ions may have a positive effect on some cells lines, whereas these same concentrations can have detrimental effects on other cell types such as macrophages. In this sense, the ions release profile could be adapted to achieve immunomodulatory concentrations within the first days after implantation, followed by a sustained release of ion concentrations that could induce stem cell proliferation and differentiation to promote new tissue formation. However, to improve the design of ion-releasing biomaterials, it is necessary to determine the toxicity and therapeutic thresholds for each phase of wound healing, which can be initially determined by the addition of free ions to cell culture media. Moreover, since biomaterial implantation will cause a pro-inflammatory response due to tissue damage, it is necessary to verify the anti-inflammatory potential of these ions in a pro-inflammatory environment, which has been scarcely investigated.

Therefore, here we aim to determine the therapeutic non-toxic concentrations of three different divalent ions that are able to guide macrophage response into an anti-inflammatory M2-like phenotype. Furthermore, their potential to mitigate different intensities of an adverse inflammatory stimulus will be assessed with the simultaneous addition of the ion, which to our knowledge, has not been previously tested.

## Results

### Macrophage metabolic activity

**Figure 1:**
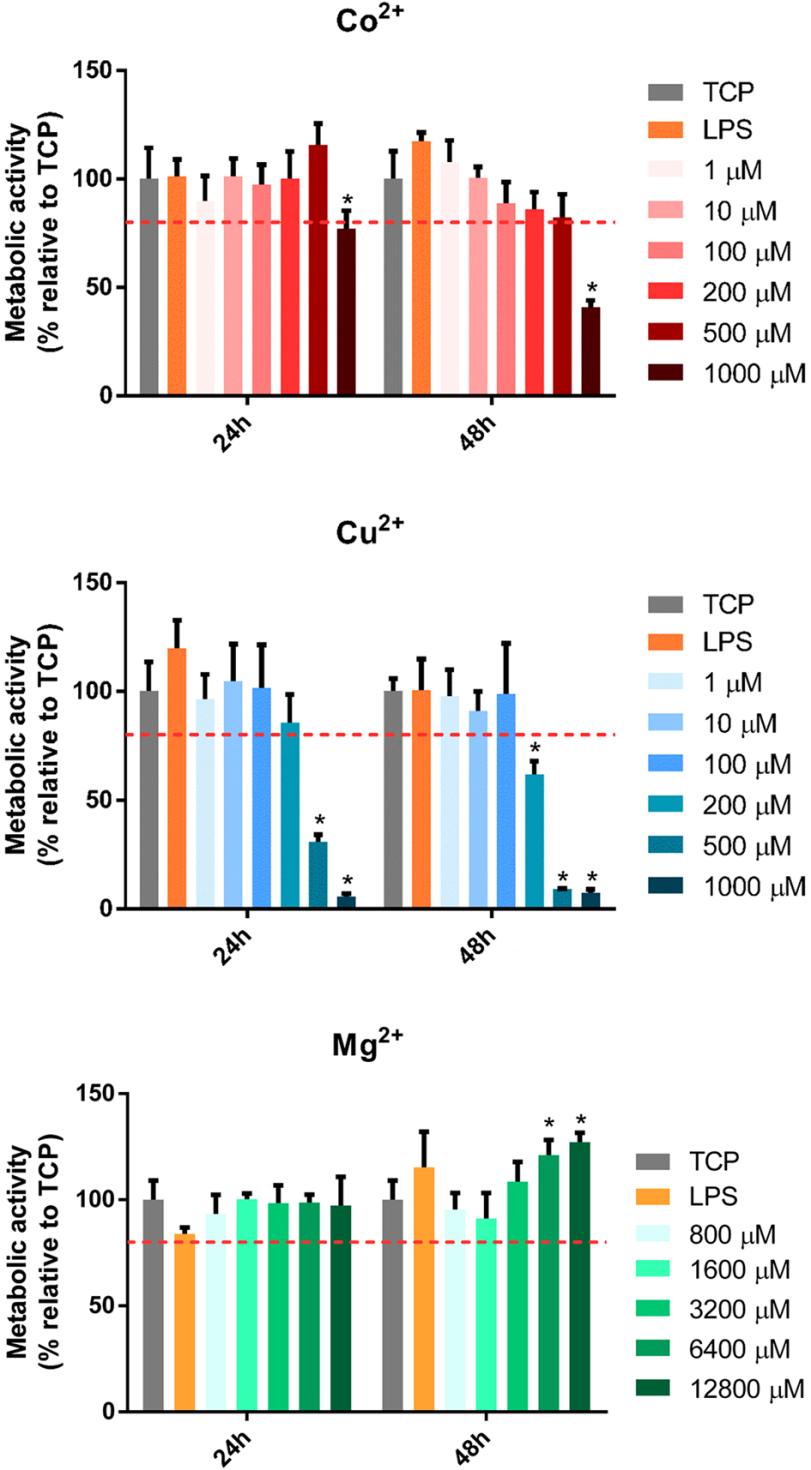
Effect of Co^2+^, Cu^2+^ and Mg^2+^ ions on THP-1 cell metabolic activity. Metabolic activity of THP-1 cells cultured with different Co^2+^, Cu^2+^ and Mg^2+^ concentrations for 24 and 48 h. Metabolic activity results were represented as percentage relative to an unstimulated control (TCP) and compared with the TCP of each day. Values lower than 80% metabolic activity (red line) which were significantly different (*p* < 0.05) from TCP were considered cytotoxic. Statistically significant differences (*p* < 0.05) were represented with *.

To determine the cytotoxicity of different Co^2+^, Cu^2+^ and Mg^2+^ concentrations, macrophage metabolic activity was analyzed and compared to an unstimulated control cultured in tissue culture plastic (TCP) (Fig. 1). Metabolic activities lower than 80% compared to the TCP were considered cytotoxic. On the one hand, Co^2+^ concentrations higher than 500 μM significantly decreased cell metabolic activity at both 24 and 48 h (*p* < 0.05). On the other hand, Cu^2+^ significantly decreased cell metabolic activity when concentrations higher than 500 μM (*p* < 0.05) at 24 h and higher than 200 μM at 48 h (*p* < 0.05). Mg^2+^ was not cytotoxic in any of the concentrations analyzed since metabolic activity did not significantly decrease. Interestingly, 6400 μM and 12,800 μM concentrations induced a significant increase in metabolic activity at 48 h.

### DNA quantification analysis

**Figure 2:**
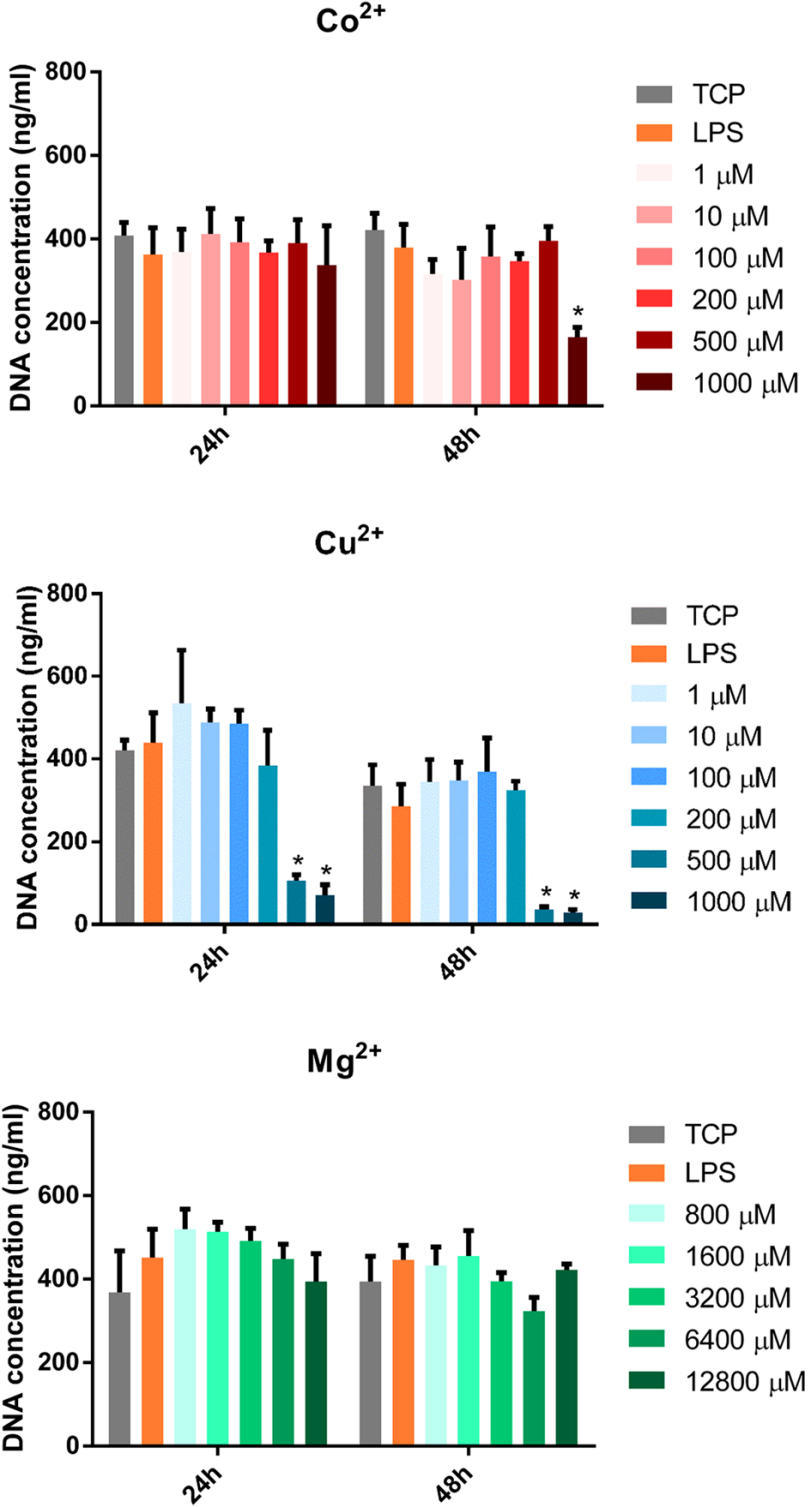
Effect of Co^2+^, Cu^2+^ and Mg^2+^ ions on THP-1 cell proliferation. DNA quantification of THP-1 cells cultured with different Co^2+^, Cu^2+^ and Mg^2+^ concentrations for 24 and 48 h. The results of DNA quantification were compared to the TCP of each day and a statistically significant (*p* < 0.05) decrease was observed in some concentrations of Co^2+^ and Cu^2+^. Statistically significant differences (*p* < 0.05) were represented with *.

To corroborate the results obtained from metabolic activity, dsDNA was quantified and compared to the TCP at each time point (Fig. 2). Co^2+^ concentrations higher than 500 μM significantly decreased dsDNA at 48 h (*p* < 0.05), confirming the cytotoxic effect observed in the metabolic activity assay. However, Cu^2+^ significantly decreased dsDNA from 500 μM at 24 and 48 h. Regarding dsDNA quantification when macrophages were cultured in presence of Mg^2+^, none of the concentrations that were analyzed induced a significant change in dsDNA quantification. A slight reduction in dsDNA was observed comparing between 24 and 48 h in all of the conditions (controls and cells cultured with the ions).

### Macrophage gene expression analysis

**Figure 3:**
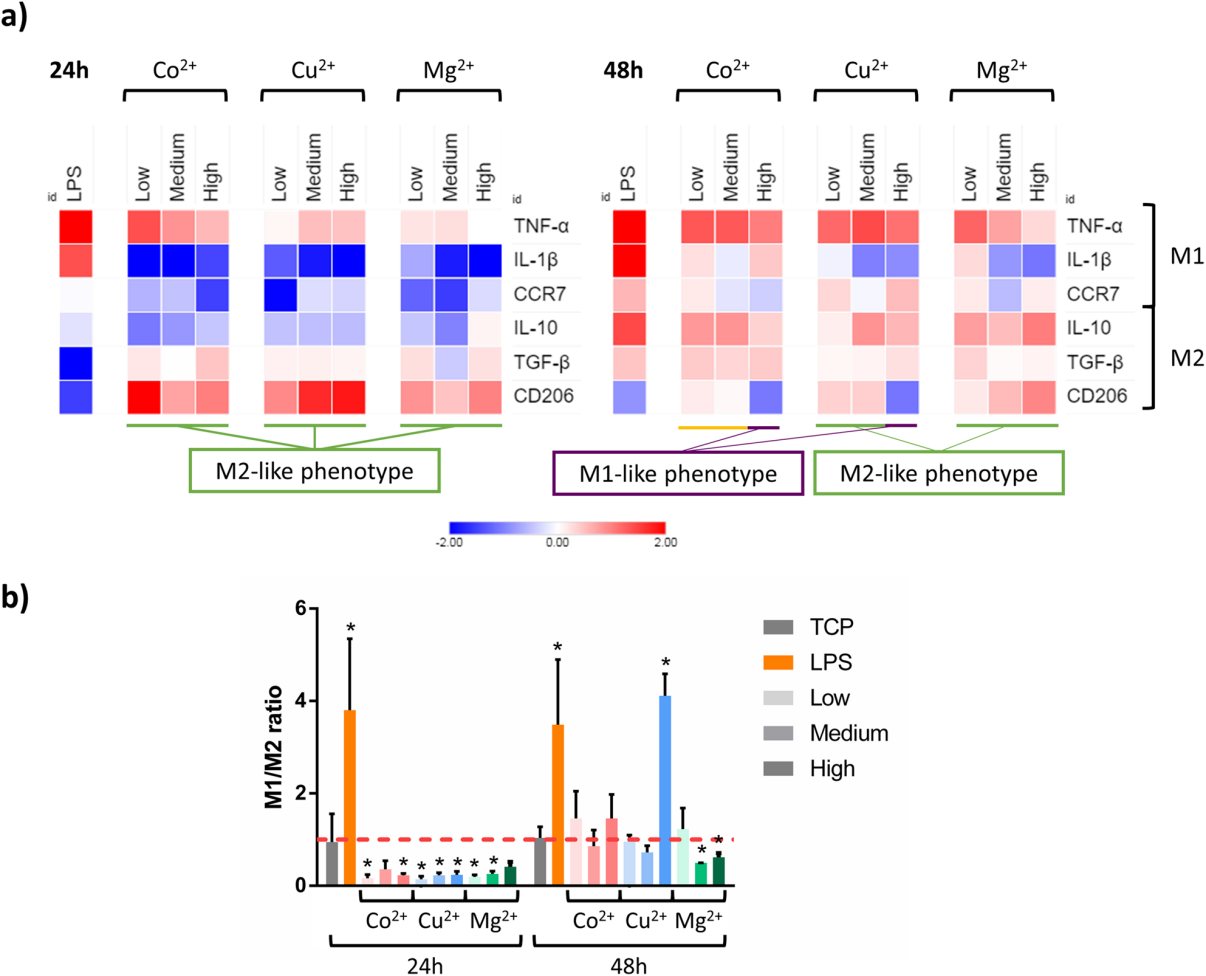
Effect of increasing concentrations of cobalt, copper and magnesium on the expression of M1 and M2 markers in THP-1 macrophage cell line. (**a**) After 24 or 48 h of incubation, the mRNA levels of three M1 markers (TNF-α, IL-1β and CCR7) and three M2 markers (IL-10, TGF-β and CD206) were determined by RT-qPCR. The purple line indicates those conditions that induce a M1-like phenotype, while the green line indicates those that induce a M2-like phenotype. Those conditions that induced a phenotype that should be further confirmed are indicated by the yellow line. (**b**) M1/M2 ratio was calculated from the gene expression of CCR7 and CD206 markers. A higher intensity of the color indicates a higher concentration of the ion. The results of the M1/M2 ratio for each condition were compared to the TCP of each day. Statistically significant differences (*p* < 0.05) were represented with *. Statistical analysis of qRT-PCR results is in Supplementary Tables S1 and S2.

THP-1 macrophages were stimulated with low, medium and high non-cytotoxic concentrations of Co^2+^, Cu^2+^ and Mg^2+^ as detailed in Table 1. Since Cu^2+^ and Co^2+^ have similar pathways in the angiogenic response, the same non-toxic concentrations were selected for both ions. At 24 and 48 h, gene expression of M1 (CCR7, TNF-α and IL-1β) and M2 (CD206, IL-10 and TGF-β) phenotype markers were determined by qRT-PCR (Fig. 3a, Supplementary Tables S1 and S2). The results were compared to the gene expression of the TCP to establish the statistically significant differences between ion stimulated and unstimulated macrophages.

**Table 1:** Summary of the conditions analyzed by gene expression.

Ion	Condition	Final concentration in cell culture medium (μM)
Co^2+^	Low	1
	Medium	10
	High	100
Cu^2+^	Low	1
	Medium	10
	High	100
Mg^yy+^	Low	800
	Medium	3200
	High	12,800

At 24 h, the stimulation of THP-1 macrophages with Co^2+^ induced an overall decrease in M1 related markers. Gene analysis showed a significant decrease in IL-1β and CCR7, while showing an increase in TNF-α expression (*p* < 0.05). Regarding the markers related with M2 polarization, low and high concentrations stimulated CD206 expression, while a decrease in IL-10 was observed in low and medium concentrations. At 48 h, low and medium Co^2+^ concentrations did not induce any significant change in CCR7 and CD206, although promoted TNF-α, IL-10 and TGF-β expression. However, high Co^2+^ concentration promoted a M1-like phenotype expression, decreasing CD206 expression and overexpressing TNF-α.

When macrophages were stimulated with Cu^2+^, the gene expression profile at 24 h was similar to that observed when stimulating with Co^2+^. At 48 h, low and high Cu^2+^ concentrations induced the expression of M1 related genes TNF-α and CCR7, respectively. Medium concentrations significantly decreased IL-1β expression (*p* < 0.05). Regarding the expression of M2 related genes, low and medium concentrations promoted CD206 expression, while medium concentrations upregulated IL-10.

Lastly, stimulation with Mg^2+^ for 24 h decreased the expression of M1 related markers IL-1β and CCR7, while stimulating M2 marker CD206. At 48 h, medium and high Mg^2+^ concentrations decreased IL-1β. CD206 expression was increased when cells were stimulated with medium and high Mg^2+^ concentrations. Moreover, an overall increase in IL-10 was observed, while only low Mg^2+^ concentration promoted an increase of TGF-β marker.

Then, as a first approach to classify the macrophage phenotype induced by Co^2+^, Cu^2+^ and Mg^2+^, M1/M2 ratio was analyzed (Fig. 3b). Values of M1/M2 ratio higher than 1 (which is the value of the TCP) were an indicator of M1 phenotype, while values lower than 1 were associated to an M2 phenotype. At 24 h, lipopolysaccharide (LPS) was able to induce a significant increase (*p* < 0.05), while all of the conditions containing ions were able to significantly decrease this ratio below 1 (*p* < 0.05), with the exception of medium Co^2+^ and high Mg^2+^ concentrations. However, only LPS and high concentrations of Cu^2+^ were able to increase the ratio above 1 at 48 h, while medium and high Mg^2+^ concentrations significantly decreased the ratio below 1 (*p* < 0.05).

### Macrophage morphology analysis

**Figure 4:**
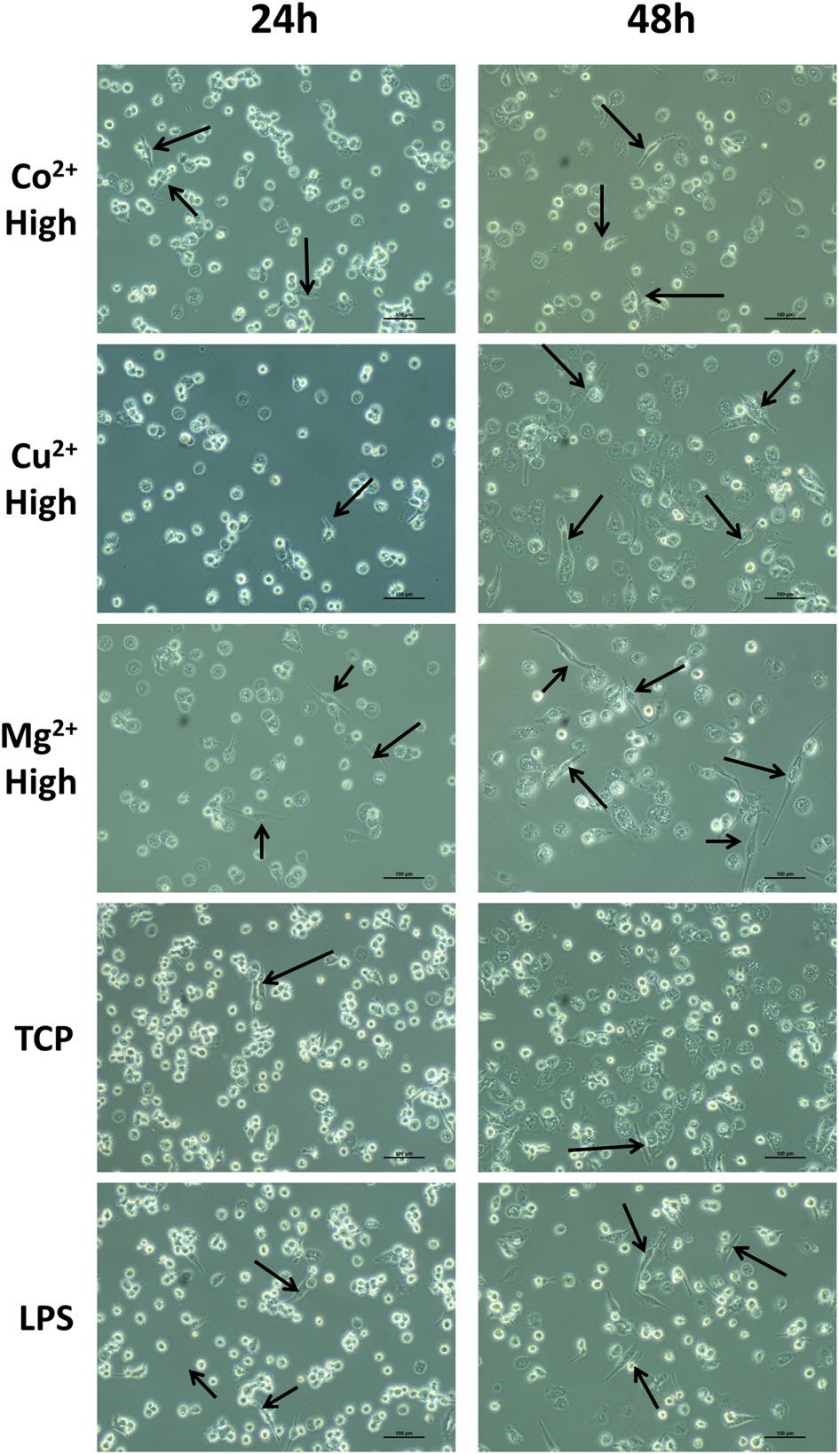
Effect of high concentrations of cobalt, copper and magnesium ions on THP-1 cell morphology. Elongated cells, which were defined as those in which the aspect ratio was higher than 2.5, are indicated with black arrows. Scale bars = 100 μm.

**Figure 5:**
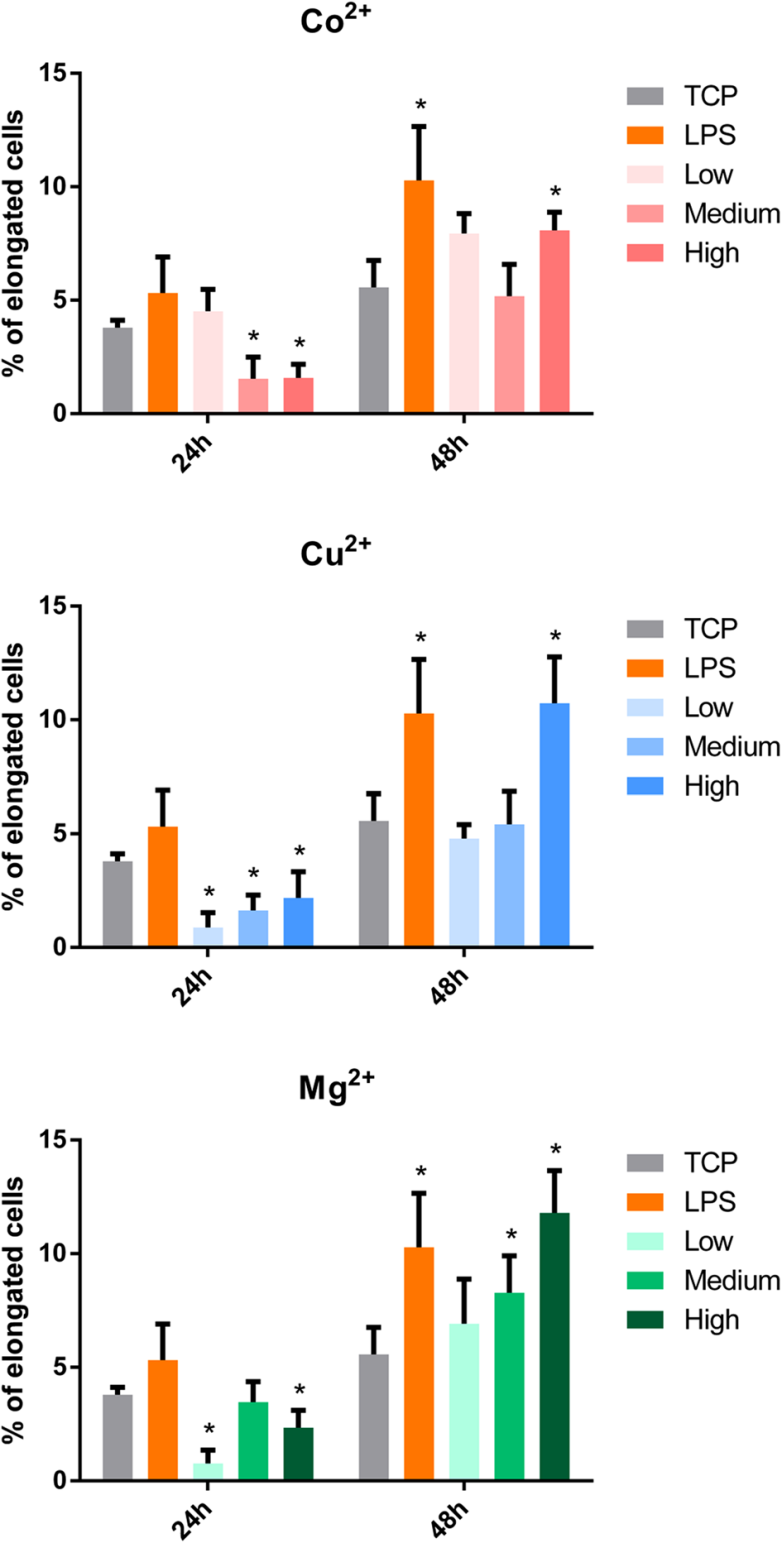
Quantitative analysis of THP-1 cell morphology. The number of elongated cells (aspect ratio higher than 2.5) in each condition was represented. Statistical significance was accepted at *p* < 0.05 and represented with *. The correlation analysis between cell elongation and gene expression can be found in Supplementary Table S3.

Phase contrast microscopy analysis was used to assess the effect of low, medium and high Co^2+^, Cu^2+^ and Mg^2+^ concentrations on cell morphology (Fig. 4, Supplementary Figure S1). Most of macrophages adopted a rounded morphology with some elongated cells, independently of the ion concentration and culture time. However, the quantification of elongated cells (Fig. 5) revealed that high Co^2+^ concentrations induced a significant reduction of elongated cells at 24 h compared to TCP (*p* < 0.05), while stimulating macrophages with this Co^2+^ concentration for 48 h induced the opposite effect compared to TCP. Cu^2+^ induced an overall decrease in the percentage of elongated cells at 24 h, while high concentrations significantly induced macrophage elongation (*p* < 0.05) at 48 h. Lastly, low and high Mg^2+^ concentrations significantly reduced elongated cells at 24 h. In contrast, medium and high concentrations increased the number of elongated cells at 48 h. Correlating the percentage of elongated cells in each condition with the expression of each gene showed a lack of significant relation (*p* > 0.05) between both of them (Supplementary Table S3).

### Mitigating the effect of a pro-inflammatory stimulus with ions

A combination of the ions and a pro-inflammatory stimulus (LPS) was used to observe if the ions were able to reverse the expression of M1 markers promoted by LPS when stimulating THP-1 macrophages. Based on our initial results, we selected medium and high Mg^2+^ concentrations since these conditions were able to reduce the M1/M2 ratio at 24 and 48 h. We also selected low and medium Cu^2+^ concentrations as they were able to induce the classical M2 markers CD206 and IL-10 while decreasing the expression of IL-1β, even though the M1/M2 ratio remained unchanged compared to the TCP. Moreover, two different aggressions with LPS (100 ng/ml for 48 h or 10 ng/ml for 24 h) were used to induce a strong or a mild pro-inflammatory response.

**Figure 6:**
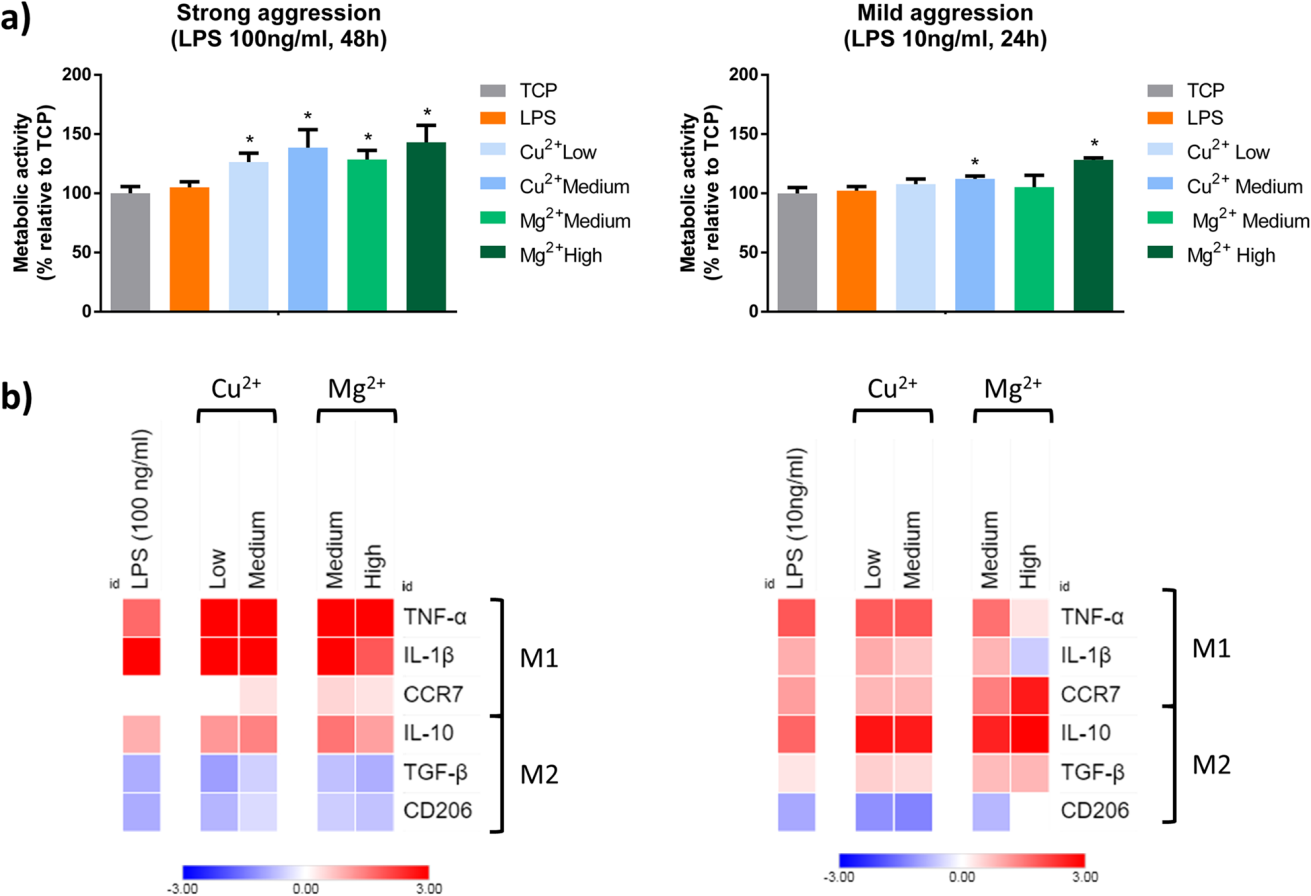
Effect of the combination of LPS with Cu^2+^ and Mg^2+^ on THP-1 cell metabolic activity (**a**) and gene expression of M1 and M2 markers (**b**). THP-1 cells were stimulated with a combination of Cu^2+^ and Mg^2+^ and 100 ng/ml LPS for 48 h (strong pro-inflammatory stimulation) or 10 ng/ml LPS for 24 h (mild pro-inflammatory stimulation). (**a**) Metabolic activity results were represented as percentage relative to an unstimulated control (TCP) and compared with the TCP of each day. Statistical significance was accepted at *p* < 0.05 and represented with *. (**b**) M1 markers (TNF-α, IL-1β and CCR7) and M2 markers (IL-10, TGF-β and CD206) were determined by RT-qPCR. Results were normalized against β-actin mRNA and are expressed relative to the mRNA levels of unstimulated THP-1 macrophages. Statistical analysis is in Supplementary Tables S4, S5, S6 and S7.

Although cell morphology changed depending on the LPS concentration, phase contrast microscopy analysis demonstrated that most macrophages adopted a rounded morphology with some elongated cells, independently of the ion concentration (Supplementary Figure S2). Cellular metabolic activity was analyzed performing a resazurin reduction assay (Fig. 6a). In both aggressions, metabolic activity increased compared to the unstimulated control, with the exception of low Cu^2+^ and medium Mg^2+^ concentrations in presence of a mild aggression. Next, the expression of M1 and M2 markers were analyzed by RT-qPCR. When THP-1 macrophages were activated with a high concentration of LPS (100 ng/ml) for 48 h in combination with Cu^2+^ and Mg^2+^, the gene expression profile corresponded to the M1 phenotype (Fig. 6b, Supplementary Tables S4 and S5). Indeed, the combination of these ions with LPS induced an upregulation of TNF-α and IL-10 expression and only high concentrations of Mg^2+^ induced a significant decrease in IL-1β expression compared to LPS alone.

When a milder aggression was performed, reducing the concentration of LPS to 10 ng/ml and the exposure time to 24 h, the presence of medium Cu^2+^ concentrations induced a significant decrease in IL-1β compared to LPS alone (Fig. 6b, Supplementary Table S6). However, the expression of other genes did not differ from the expression obtained when cells were stimulated only with LPS (Fig. 6b, Supplementary Table S7). Comparatively, the combination of high Mg^2+^ with LPS significantly downregulated the expression of TNF-α and IL-1β while raising the levels of CD206 compared to LPS alone (Fig. 6b, Supplementary Table S7). When these results were compared to an unstimulated control, it was observed that the expression of TNF-α and CD206 was not significantly different from the control (Fig. 6b, Supplementary Table S6).

## Discussion

In the present study, we investigated the effect of Co^2+^, Cu^2+^ and Mg^2+^ on macrophage polarization, analyzing the gene expression of M1 and M2 markers and cell morphology in THP-1 human monocytic cell line. This study was performed to establish possible therapeutic concentrations of these ions that could modulate macrophage response and induce a M2-like phenotype. Moreover, we investigated the potential of Cu^2+^ and Mg^2+^ to modulate the inflammatory response caused by a pro-inflammatory stimulus such as LPS, simulating a clinical scenario in which the disruption caused by a device implantation will start an inflammatory response.

First, we determined the cytotoxicity of each ion concentration at 24 and 48 h of cell culture, assessing cell metabolic activity and DNA concentration (Figs. 1 and 2). On the one hand, Co^2+^ concentrations lower than 1000 μM did not have a cytotoxic effect, as observed when the metabolic activity and DNA concentration was quantified. On the other hand, Cu^2+^ concentrations equal or higher than 200 μM caused a significant reduction in metabolic activity, while concentrations higher than 500 μM caused a reduction in DNA concentration. Mg^2+^ did not produce any significant reduction in cell viability when THP-1 macrophages were stimulated with concentrations until 12,800 μM, in accordance with a previous study^24^. However, Mg^2+^ concentrations higher than 3200 µM were able to induce an increase in cells’ metabolic activity. The metabolic activity assay is based on the intracellular reduction of resazurin to resorufin in metabolically active cells, mainly by mitochondrial enzymes and enzymes in the respiratory chain which use NADH and NADPH as electron donors^25^. Previous reports showed that Co^2+^ and Cu^2+^ are able to impair mitochondrial activity, which is related to a decrease in cell viability. For example, Co^2+^ increased the expression of proteins related to the mitochondrial pathway of apoptosis, as well as oxidative stress^26,27^. However, Cu^2+^ impaired the function of the electron transfer and the functionality of redox sensitive proteins in the mitochondria, causing a cytotoxic effect^28^. Therefore, the cytotoxic effect observed in presence of Co^2+^ and Cu^2+^ happens due to a dysfunction of the mitochondrial function. In contrast, the Mg^2+^ induced increase in the metabolic activity could be explained by the increase in the number of mitochondria, as suggested by previous reports^29^.

Once the cytotoxic levels of the different ions were determined, we investigated the genetic profile that Co^2+^, Cu^2+^ and Mg^2+^ induced to THP-1 macrophages (Fig. 3). Gene expression analysis of THP-1 macrophages stimulated with increasing non-cytotoxic Co^2+^ concentrations (1, 10 and 100 μM) yielded different expression profiles depending on the concentration and stimulation time. Thus, the increase of CD206 marker expression combined with the decrease in CCR7 and IL-1β at 24 h suggest that, at first, macrophages stimulated with Co^2+^ induced a M2-like phenotype, which could also be observed when the M1/M2 ratio was analyzed. However, the M1/M2 ratio was unable to indicate significant differences in the phenotype when a longer stimulation with Co^2+^ was performed. As a consequence, the expression of other M1 and M2 markers was observed to elucidate the macrophage phenotype. Longer stimulations with low and medium (1 and 10 μM) concentrations of Co^2+^ induced a phenotype which expresses TNF-α and IL-10 without altering CD206 levels. It has been previously described that some subtypes of M2 phenotype are able to express both TNF-α and IL-10, so these results could be indicating the presence of an M2-like phenotype^30,31^. In fact, macrophage stimulation with 10 μM Co^2+^ concentration has created controversy in the literature. Several studies reported that stimulating with 10 μM Co^2+^ increased IL-1β^32^ and TNF-α release^33^. Overall, these studies suggested that macrophages stimulation with Co^2+^ generates a pro-inflammatory environment. However, previous studies did not measure the levels of M2 marker IL-10, which can be used to distinguish between M1 and M2 phenotypes. Thus, our results indicate that low and medium Co^2+^ concentrations might be able to induce an M2-like phenotype, but further studies such as the detection of M2 subtype specific markers should be performed to confirm this fact.

Stimulating macrophages with higher concentrations of Co^2+^ for 48 h promoted the expression of markers corresponding to an M1-like phenotype, increasing TNF-α and reducing CD206 gene expression. Previous studies have described that 100 μM Co^2+^ increased the expression and release of pro-inflammatory cytokines such as TNF-α and IL-6^20,34^. Moreover, another study observed that this Co^2+^ concentration induced the activation of NFκB transcription factor, which is mainly responsible for the inflammatory response in macrophages^20^. Further studies have described that high concentrations of Co^2+^ activate PI3K/Akt pathway, which increases the expression of membrane receptor TLR4, associated to M1 phenotype^35^. The results of our study indicate that long term stimulation of macrophages with high Co^2+^ concentrations promote macrophage polarization towards an M1-like phenotype.

Cu^2+^ induced a similar gene expression profile at 24 h compared to Co^2+^, suggesting that initially they might induce a M2-like phenotype regardless of the concentration used. This fact corresponded with the analysis of the M1/M2 ratio. At 48 h, low and medium Cu^2+^ concentrations were able to maintain the expression of M2 related markers. However, higher Cu^2+^ concentrations (100 μM) caused a significant decrease in CD206 while maintaining an upregulated expression of TNF-α, indicating a switch in macrophage phenotype to M1, which was further confirmed when analyzing the M1/M2 ratio. This condition also promoted the expression of IL-10, which could be a regulatory response similar to the one observed with the positive control of inflammation LPS^36^. The role of copper ions on macrophage polarization has been poorly investigated, and the studies have been conducted using copper-loaded biomaterials which have yielded interesting results. For example, the release of Cu^2+^ concentrations of 1.5 mg/L (23.6 μM) released from silica nanospheres increased the expression of pro-inflammatory markers such as CCR7, TNF-α, IL-1β, IFN-γ and IL-6^21^. A similar expression profile was observed when macrophages were seeded onto a copper containing bioceramic coating on a titanium substrate. In this case, the expression of the pro-inflammatory markers iNOS and NFκB were upregulated and pro-inflammatory cytokines were upregulated when releasing 12 and 22 μM copper ions^37^. However, when lower concentrations (between 1 and 5 μM) of copper ions were released from a copper doped Ti6Al4V alloy the expression of pro-inflammatory markers IL-1β, IL-6, TNF-α and CCR7 were downregulated and increased the expression of IL-10 and CD206^22,38^. These results, combined with our findings, indicate that Cu^2+^ concentrations equal or lower than 10 μM are able to induce macrophage polarization towards an M2 phenotype, while higher concentrations stimulate a pro-inflammatory M1 response.

Regarding the response promoted by Mg^2+^, the increase in CD206 and the decrease in CCR7 and IL-1β observed at 24 h suggested that these ions could be stimulating a M2-like response, which was still maintained at 48 h, especially when macrophages were stimulated with medium and high (3200 and 12,800 μM) Mg^2+^ concentrations, which induced CD206 and IL-10 expression while decreasing the levels of IL-1β. These results are in accordance with a previous study in which cell culture media supplemented with 0.4 and 20 mM Mg^2+^ promoted an increase in the expression of CD206 and IL-10, while decreasing pro-inflammatory markers such as CCR7 and TNF-α^23^. Moreover, the release of magnesium ions from certain biomaterials such as magnesium microparticles has also promoted macrophage polarization towards an M2 phenotype^39^. In these studies, released concentrations ranging from 2 to 11.14 mM reduced pro-inflammatory cytokine expression. Furthermore, magnesium microparticles and magnesium doped titanium increased the expression of IL-10 and CD206. Thus, our results confirm the potential of magnesium ions to induce M2-like polarization in macrophages.

To further characterize macrophage response to Co^2+^, Cu^2+^ and Mg^2+^ ions, macrophage morphology was analyzed and correlated to the gene expression (Fig. 5 and Supplementary Table S3), since it has been previously described that macrophage morphology could be an indicator of macrophage phenotype. Previous results have shown that under specific stimuli, M1 macrophages exhibited a round shape, while M2 macrophages appeared with a spindle-like shape^9,40,41^. However, no correlation between M1 or M2 gene expression and macrophage elongation was found in our study. Indeed, high Co^2+^ and Cu^2+^ concentrations were able to induce a significant increase in cell elongation at 48 h, when these conditions have been associated to M1 macrophage phenotype as discussed before. Therefore, macrophage morphology might not be a good indicator of M1 or M2 phenotypes. In fact, several studies have suggested that macrophage morphology could be stimuli dependent, rather than phenotype dependent. For example, Porcheray et al. described that macrophages stimulated with IL-10 were able to adopt an elongated morphology, while those stimulated with dexamethasone (a potent M2 phenotype inducer) exhibited a round shape^42^. Nevertheless, the physical induction of an elongated morphology using micropatterned surfaces or aligned nanofibers increased the expression of markers associated to the M2 phenotype such as IL-10 and Arginase-1^9,43,44^. This could mean that soluble cues could have an effect in the expression of M1 and M2 markers that might not be represented by cell morphology, while topological cues could actively induce changes in cell elongation that are evidenced in M2 marker expression. In this sense, the design of biomaterials that combine micropatterns that induce macrophage elongation and the release of M2 inducers could promote a synergistic effect. This combination has already been used for bone regeneration applications, where the combination of surface topographies with Cu^2+^ release improved osteogenesis. However, this approach has not been explored in the context of immunomodulation to the best of our knowledge^45^.

Next, we further tested the anti-inflammatory potential of the concentrations of Cu^2+^ and Mg^2+^ that were able to induce an M2-like phenotype by combining them with two intensities of pro-inflammatory aggression (Fig. 6). When a strong aggression was applied (100 ng/ml LPS for 48 h), low and medium Cu^2+^ and medium and high Mg^2+^ were unable to reduce the expression of pro-inflammatory markers, and even they potentiated the expression of TNF-α. However, when macrophages were exposed to a mild aggression (10 ng/ml LPS for 24 h), Cu^2+^ maintained the expression of pro-inflammatory markers, while high Mg^2+^ concentrations reduced significantly the expression of pro-inflammatory markers, TNF-α and IL-1β. Moreover, this concentration of magnesium potentiated IL-10 and TGF-β expression, while increasing CD206 levels compared to LPS-activated macrophages. These findings indicate that high concentrations of Mg^2+^ can modulate the pro-inflammatory response caused by a mild aggression with LPS. In both aggressions, metabolic activity was increased, which might be a consequence of a dysregulation of mitochondrial metabolism. It has been previously described that the differentiation of macrophages to M1 phenotype can have an effect in mitochondrial metabolism, which is responsible for resazurin reduction to resorufin^46^. However, to the best of our knowledge, the mitochondrial pathways in the presence of anti-inflammatory mediators such as Mg^2+^ in combination with LPS has not been described yet.

**Figure 7:**
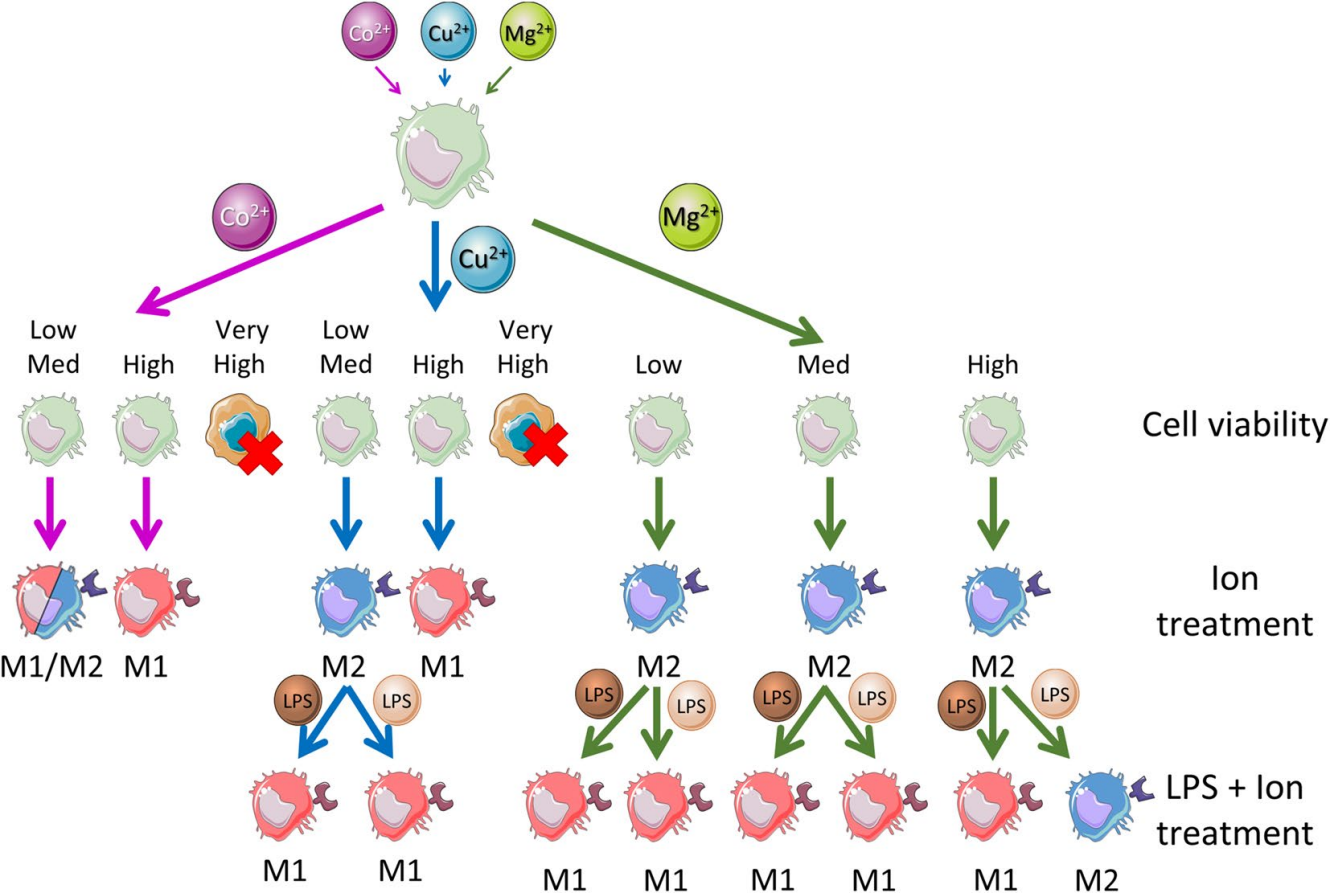
Schematic illustration of the macrophage phenotype promoted by Co^2+^, Cu^2+^ and Mg^2+^ bioactive ions, alone or in presence of a pro-inflammatory molecule such as LPS. Darker color of LPS represents a strong aggression, while a lighter color represents a mild aggression. This figure was produced using Servier Medical Art (http://smart.servier.com/).

The aggression of macrophages with LPS in presence of Cu^2+^ has not been profoundly described in the literature. We performed a competitive assay by adding LPS and the ions simultaneously since in a clinical situation in which an ion releasing biomaterial is implanted, the pro-inflammatory response caused by tissue damage and ion release would begin simultaneously. Previous studies described a reduction in the production of nitric oxide (pro-inflammatory molecule) while no difference was observed in TNF-α levels compared to LPS-activated macrophages when they were pre-treated with 100 μM Cu^2+^ prior to activation with 1 µg/ml LPS^28,47^. These observations confirm the tendency seen in our results, which suggests that copper ions have little or no effect in reducing the pro-inflammatory response caused by LPS. Regarding macrophage response in presence of both LPS and Mg^2+^, media supplementation with 2.5 mM Mg^2+^ significantly decreased IL-6 and TNF-α expression in LPS-activated macrophages, and reduced NFκB activation, which was further confirmed by other publications^48,49^. However, contrary to our observations, Hu et al. observed a reduction in IL-10 and CD206 markers when they treated macrophages with 5 and 10 mM Mg^2+^, 10 ng/ml LPS and 10 ng/ml IFN-γ for 24h^48^. Nevertheless, these articles confirm the general tendency observed in our results, which suggest that Mg^2+^ could inhibit the pro-inflammatory response caused by LPS when the extent of this response is limited. However, magnesium ions did not have this effect when stimulated strong aggression with LPS was performed, suggesting a limited ability of magnesium ions to induce an anti-inflammatory response. In this sense, the anti-inflammatory potential of high Mg^2+^ concentrations should be analyzed in detail in future studies, observing its biological effect in the release of inflammatory mediators, such as cytokines, reactive oxygen species or nitric oxide, among others. Figure 7 shows a schematic representation of the macrophage phentoypes observed when a treatment with ions and a combined treatment with ions and LPS was performed.

## Conclusions

Bioactive ions have been demonstrated to improve tissue regeneration. However, there are limited evidences showing the ion effect on the inflammatory phase and, therefore, we assessed the effect of different Co^2+^, Cu^2+^ and Mg^2+^ concentrations on macrophage response. Herein, we demonstrated that Co^2+^ induced a pro-inflammatory phenotype, while Cu^2+^ and Mg^2+^ stimulated the expression of anti-inflammatory markers in a dose dependent manner. However, only 12,800 μM Mg^2+^ showed certain anti-inflammatory effect under a mild pro-inflammatory aggression of macrophages. Our results indicate that Cu^2+^ and Mg^2+^ ions can be used to modulate macrophage response; however, only Mg^2+^ will have limited efficiency in a pro-inflammatory scenario.

## Materials and methods

### Cell culture and differentiation

THP-1 monocytic cell line was obtained from DSMZ (ACC 16) and maintained at 3 × 10^5^ cells/ml in RPMI 1640 medium (Sigma) supplemented with fetal bovine serum (10%, FBS, Sigma) and penicillin–streptomycin (1%, Fisher Scientific). To differentiate THP-1 cells into macrophages, 3 × 10^4^ cells/cm^2^ were seeded and exposed to phorbol 12-myristate 13-acetate (PMA, Sigma) at a final concentration of 10 ng/ml for 8 h. Newly differentiated macrophages were washed with DPBS and incubated with cell media that contained 0–1000 μM concentrations of Co^2+^ and Cu^2+^ (cobalt(II) chloride hexahydrate and copper(II) chloride dihydrate, Sigma) and 800–12,800 μM concentrations of Mg^2+^ (magnesium chloride, Sigma) for 24 and 48 h at 37 °C, 5% CO_2_ and 95% humidified air. In parallel to the cell culture with the ions, cells were stimulated with 100 ng/ml LPS (Sigma) for 24 and 48 h as a positive control of a pro-inflammatory stimulus. Moreover, cells cultured in absence of ions were used as a negative control of inflammation (TCP).

### Macrophage metabolic activity and proliferation analysis

Metabolic activity was assessed performing a resazurin reduction assay. Cells were incubated for 3 h at 37 °C and 5% CO2 with 200 µl of cell culture medium containing 10 µg/ml resazurin sodium salt (Sigma) at a final concentration of 10 µg/ml. Then, 100 µl were transferred to a 96-well plate and absorbance was measured at 570 and 600 nm using a microplate reader (Synergy HT, Bio-Tek). Cell metabolic activity was expressed in terms of % reduction of resazurin and then normalized to control values obtained from unstimulated cells in tissue culture plastic (TCP). dsDNA was quantified using Quant-iT™ PicoGreen™ dsDNA Assay Kit (Invitrogen) following the manufacturer’s guidelines. Briefly, cells were washed with sterile DPBS and frozen with 200 µl of TE1X. Three freeze thaw cycles were performed to lyse the cells. Then, 100 µl of the thawed samples were mixed with 100 µl of a 1:200 dilution of PicoGreen reagent in a black 96 well plate (Greiner) and incubated for 5 min RT in the dark. Finally, fluorescence was measured at an excitation wavelength of 480 nm and an emission wavelength of 520 nm.

### Macrophage gene expression analysis

Gene expression of specific macrophage markers was evaluated by qRT-PCR. Total RNA from THP-1 cells was isolated with Nucleospin® RNA/protein kit (Macherey–Nagel) following the manufacturer’s instructions. Quantification of extracted RNA was performed using a microvolume plate (Take3, Bio-Tek) to measure absorbance in a microplate reader. cDNA synthesis was performed using Transcriptor cDNA Synthesis Kit (Roche) and following the manufacturer’s protocol with anchored oligo(dT)_18_ primers and random hexamer primers. To detect target mRNAs, quantitative PCR was performed with QuantiNova SYBR Green PCR Kit (Qiagen). Briefly, 10 ng of cDNA per reaction were amplified performing an initial activation step of 2 min at 95 °C, denaturation for 5 s at 95 °C and 40 cycles of annealing/extension for 10 s at 60 °C in a CFX96 Real-Time System (BioRad). Pro-inflammatory markers (M1) analyzed were CCR7, TNF-α and IL-1β, while anti-inflammatory (M2) markers were CD206, IL-10 and TGF-β. The primers of the target genes are detailed in Supplementary Table S8. Expression of genes was normalized using the mean threshold cycle (Ct) value of the housekeeping gene β-actin. The 2^−ΔΔCt^ method was used to compare the mRNA expression levels between different groups. Then, the results of fold expression were transformed in log2 data and the mean values were represented in heatmaps using Morpheus matrix visualization and analysis software. Moreover, the M1/M2 ratio was calculated from the results of CCR7 and CD206 expression, as described elsewhere^6^. This ratio can be used to determine the overall proportion of cells of each phenotype. Values of the M1/M2 ratio greater than 1 indicate that the predominant phenotype is M1, while values lower than 1 indicate that the predominant phenotype is M2.

### Macrophage morphology analysis

The morphology of differentiated THP-1 macrophages was analyzed by optical microscopy. Pictures were taken using an Olympus CKX41 microscope equipped with a Nikon DS-Fi1 camera. Elongated cells and total amount of cells were quantified using ImageJ 1.52p software (National Institute of Health), counting 5 different regions per condition and at least 100 cells per region. Cell morphology was determined analyzing the aspect ratio of each cell, which compares the length of major axis with the length of minor axis of a cell. As previously reported, aspect ratios higher than 2.5 were associated to elongated cells^10^.

### Mitigating the effect of a pro-inflammatory stimulus with ions

Ion concentrations that induced an M2 macrophage phenotype were then tested in a pro-inflammatory condition, using LPS-stimulated macrophages. Macrophages were simultaneously exposed to two intensities of LPS stimulation, in combination with the ions: a mild aggression with 10 ng/ml LPS for 24 h or a strong aggression with 100 ng/ml LPS for 48h^50,51^. Macrophage response was characterized by cell morphology, metabolic activity and gene expression following the methods described above.

### Statistical analysis

Metabolic activity and dsDNA quantitation assays were carried out in quadruplicate, while gene expression was performed in triplicate. Data are expressed as mean ± standard deviation. The statistical analysis was performed using MINITAB^®^ (version 18, Minitab Inc.). Nonparametric statistics were used as normal distribution from each sample population (Anderson–Darling normality test) was confirmed (*p* > 0.05); however, the equality of variances (Bartlett’s and Levene’s tests for homogeneity of variance) was not confirmed (*p* < 0.05). Consequently, Kruskal–Wallis for multiple comparison analysis and Mann–Whitney for one to one comparison were carried out. Moreover, Spearman’s correlation analysis was performed on the proportion of elongated cells compared to the gene expression levels of each marker. Statistical significance was accepted at *p* < 0.05.

## Supplementary Information

Supplementary Information.

## Data Availability

The datasets generated and analysed during the current study are available from the corresponding author on reasonable request.
