# Supplementary material for: Evaluation of the immunomodulatory effects of cobalt, copper and magnesium ions in a pro inflammatory environment
Source: Sci Rep. 2021 Jun 3;11:11707. doi: 10.1038/s41598-021-91070-0 (PMC8175577; doi:10.1038/s41598-021-91070-0)
Supplement: Supplementary file 1 — Supplementary Information. [file 41598_2021_91070_MOESM1_ESM.docx]

**Supporting Information**

# Title

Evaluation of the immunomodulatory effects of cobalt, copper and magnesium ions in a pro inflammatory environment

# Authors

Leire Díez-Tercero^1^, Luis M Delgado^1*^, Elia Bosch-Rué^1^, Román A Pérez^1*^

# Affiliation

^1^Bioengineering Institute of Technology, Universitat Internacional de Catalunya, Sant Cugat del Vallès, Barcelona, Spain.

*Correspondence and requests for materials should be addressed to R.A.P. (email: [rperezan@uic.es](mailto:rperezan@uic.es)) and to L.M.D. (email: [lmdelgado@uic.es](mailto:lmdelgado@uic.es))

# Tables

**Supplementary Table S1.** Effect of Co^2+^, Cu^2+^ and Mg^2+^ on the expression of M1 and M2 in THP-1 macrophage cell line after 24 hours, statistically significant difference compared to tissue culture plastic (TCP) (* p<0.05, ns: not significant).

|  | | **Ion concentration (μM)** | | | | | | | | |
| --- | --- | --- | --- | --- | --- | --- | --- | --- | --- | --- |
| **Gene** | **LPS** | **Co^2+^** | | | **Cu^2+^** | | | **Mg^2+^** | | |
|  |  | **1** | **10** | **100** | **1** | **10** | **100** | **800** | **3200** | **12800** |
| TNF-α | * | * | * | * | ns | * | * | * | * | ns |
| IL-1β | * | * | * | * | * | * | * | * | * | * |
| CCR7 | ns | * | * | * | * | ns | * | * | * | ns |
| IL-10 | ns | * | * | ns | * | ns | ns | ns | * | Ns |
| TGF-β | * | ns | ns | * | ns | ns | ns | ns | ns | ns |
| CD206 | * | * | ns | * | * | * | * | * | ns | * |

**Supplementary Table S2.** Effect of Co^2+^, Cu^2+^ and Mg^2+^ on the expression of M1 and M2 in THP-1 macrophage cell line after 48 hours, statistically significant difference compared to tissue culture plastic (TCP) (* p<0.05, ns: not significant).

|  | | **Ion concentration (μM)** | | | | | | | | |
| --- | --- | --- | --- | --- | --- | --- | --- | --- | --- | --- |
| **Gene** | **LPS** | **Co^2+^** | | | **Cu^2+^** | | | **Mg^2+^** | | |
|  |  | **1** | **10** | **100** | **1** | **10** | **100** | **800** | **3200** | **12800** |
| TNF-α | * | * | * | * | * | * | * | * | * | ns |
| IL-1β | * | ns | ns | ns | ns | * | * | ns | * | * |
| CCR7 | * | ns | ns | ns | ns | ns | ns | ns | * | ns |
| IL-10 | * | * | * | ns | ns | * | * | * | * | * |
| TGF-β | * | * | * | * | ns | ns | ns | * | ns | ns |
| CD206 | * | * | ns | * | * | * | * | * | ns | * |

**Supplementary Table S3**. Correlation analysis of M1 (CCR7, TNF-α and IL-1β) and M2 (CD206, IL-10 and TGF-β) gene expression and the number of elongated. Cells showed a lack of relation between cell elongation and macrophage phenotypes at 24 and 48h. Spearman correlation coefficients and level of significance (P-value) are presented.

| **Fold expression vs elongated cells** | **24h** | | **48h** | |
| --- | --- | --- | --- | --- |
| **Gene** | **Correlation coefficient** | **P-value** | **Correlation coefficient** | **P-value** |
| TNF-α | 0,273 | 0,417 | -0,255 | 0,450 |
| IL-1β | 0,109 | 0,750 | -0,100 | 0,770 |
| CCR7 | 0,527 | 0,096 | 0,191 | 0,574 |
| IL-10 | 0,036 | 0,915 | 0,336 | 0,312 |
| TGF-β | -0,509 | 0,110 | -0,109 | 0,750 |
| CD206 | -0,164 | 0,631 | 0,200 | 0,555 |

**Supplementary Table S4.** Effect of the combination of LPS with Cu^2+^ and Mg^2+^ on THP-1 gene expression of M1 and M2 markers under a strong stimulation (100 ng/ml LPS for 48 hours). Statistically significant difference compared to tissue culture plastic (TCP) (* p<0.05, ns: not significant).

|  | | **Cu^2+^** | | **Mg^2+^** | |
| --- | --- | --- | --- | --- | --- |
| **Gene** | **LPS** | **1 μM** | **10 μM** | **3200 μM** | **12800 μM** |
| TNF-α | * | * | * | * | * |
| IL-1β | * | * | * | * | * |
| CCR7 | ns | ns | ns | ns | ns |
| IL-10 | * | * | * | * | * |
| TGF-β | * | * | * | * | * |
| CD206 | * | * | * | * | * |

**Supplementary Table S5.** Effect of the combination of LPS with Cu^2+^ and Mg^2+^ on THP-1 gene expression of M1 and M2 markers under a strong stimulation (100 ng/ml LPS for 48 hours). Statistically significant difference compared to pro-inflammatory control with lipopolysaccharide (LPS) (* p<0.05, ns: not significant).

|  | **Cu^2+^** | | **Mg^2+^** | |
| --- | --- | --- | --- | --- |
| **Gene** | **1 μM** | **10 μM** | **3200 μM** | **12800 μM** |
| TNF-α | * | * | * | * |
| IL-1β | * | ns | ns | * |
| CCR7 | ns | ns | ns | ns |
| IL-10 | * | * | * | * |
| TGF-β | ns | ns | ns | ns |
| CD206 | ns | ns | ns | ns |

**Supplementary Table S6.** Effect of the combination of LPS with Cu^2+^ and Mg^2+^ on THP-1 gene expression of M1 and M2 markers under a mild stimulation (10 ng/ml LPS for 24 hours). Statistically significant difference compared to tissue culture plastic (TCP) (* p<0.05, ns: not significant).

|  | | **Cu^2+^** | | **Mg^2+^** | |
| --- | --- | --- | --- | --- | --- |
| **Gene** | **LPS** | **1 μM** | **10 μM** | **3200 μM** | **12800 μM** |
| TNF-α | * | * | * | * | ns |
| IL-1β | * | * | * | * | * |
| CCR7 | * | * | * | * | * |
| IL-10 | * | * | * | * | * |
| TGF-β | ns | ns | ns | * | * |
| CD206 | * | * | * | * | ns |

**Supplementary Table S7.** Effect of the combination of LPS with Cu^2+^ and Mg^2+^ on THP-1 gene expression of M1 and M2 markers under a mild stimulation (10 ng/ml LPS for 24 hours). Statistically significant difference compared to pro-inflammatory control with lipopolysaccharide (LPS) (* p<0.05, ns: not significant).

|  | **Cu^2+^** | | **Mg^2+^** | |
| --- | --- | --- | --- | --- |
| **Gene** | **1 μM** | **10 μM** | **3200 μM** | **12800 μM** |
| TNF-α | ns | ns | ns | * |
| IL-1β | ns | * | ns | * |
| CCR7 | ns | ns | ns | * |
| IL-10 | * | * | * | * |
| TGF-β | ns | ns | * | * |
| CD206 | ns | ns | ns | * |

**Supplementary Table S8**. Primer sequences for qRT-PCR.

| **Genes** | **Primer Sequence (5’-3’)** |
| --- | --- |
| M1 macrophage phenotype | |
| TNF-α | Forward: TTCCAGACTTCCTTGAGACACG |
|  | Reverse: AAACATGTCTGAGCCAAGGC |
| IL-1β | Forward: GACACATGGGATAACGAGGC |
|  | Reverse: ACGCAGGACAGGTACAGATT |
| CCR7 | Forward: GGCTGGTCGTGTTGACCTAT |
|  | Reverse: ACGTAGCGGTCAATGCTGAT |
| M2 macrophage phenotype | |
| IL-10 | Forward: AAGCCTGACCACGCTTTCTA |
|  | Reverse: ATGAAGTGGTTGGGGAATGA |
| TGF-β | Forward: TTGATGTCACCGGAGTTGTG |
|  | Reverse: TGATGTCCACTTGCAGTGTG |
| CD206 | Forward: CCTGGAAAAAGCTGTGTGTCAC |
|  | Reverse: AGTGGTGTTGCCCTTTTTGC |
| Housekeeping | |
| β-actin | Forward: AGAGCTACGAGCTGCCTGAC |
|  | Reverse: AGCACTGTGTTGGCGTACAG |

# Figures


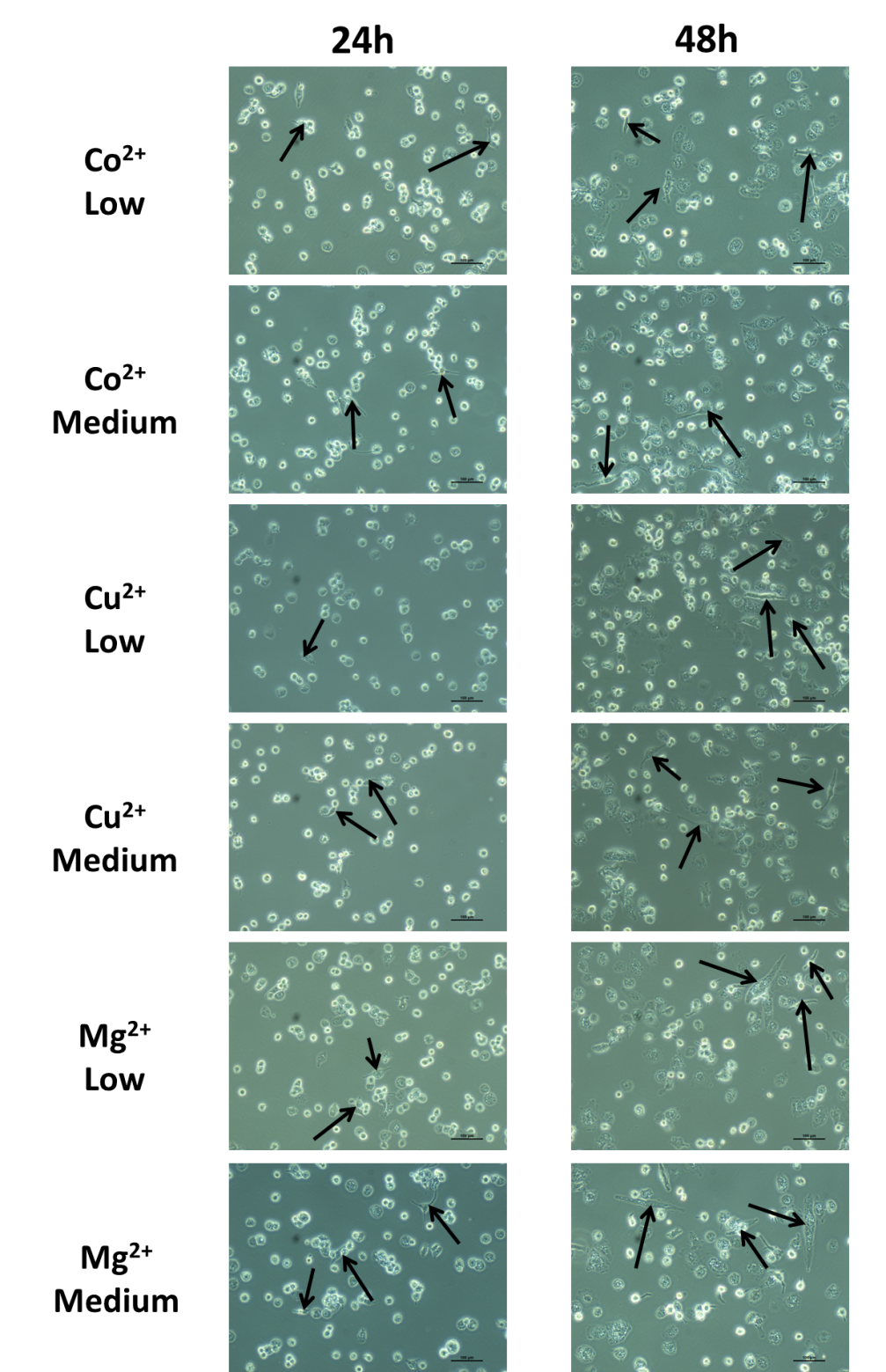


**Supplementary Figure S1.** Effect of low and medium concentrations of cobalt, copper and magnesium ions on THP-1 cell morphology. Elongated cells, which were defined as those in which the aspect ratio was higher than 2.5, are indicated with black arrows. Scale bars =100μm.


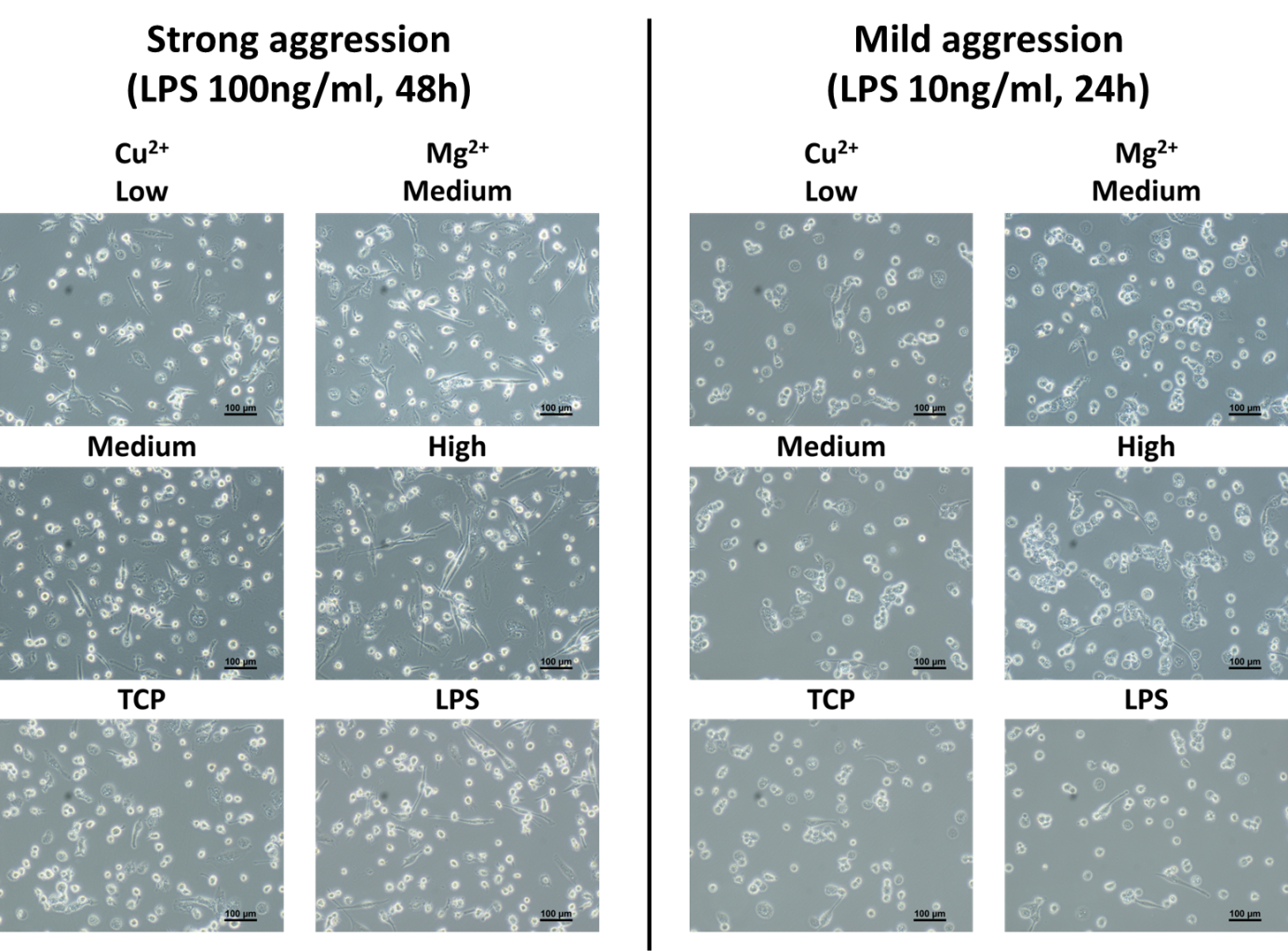


**Supplementary Figure S2.** Effect of the combination of LPS with Cu^2+^ and Mg^2+^ on THP-1 cell morphology. Scale bars =100μm
